# Comparative Genomics of Sigma Factors in *Acidithiobacillia* Sheds Light into the Transcriptional Regulatory Networks Involved in Biogeochemical Dynamics in Extreme Acidic Environments

**DOI:** 10.3390/microorganisms13061199

**Published:** 2025-05-24

**Authors:** Pedro Sepúlveda-Rebolledo, Carolina González-Rosales, Mark Dopson, Ernesto Pérez-Rueda, David S. Holmes, Jorge H. Valdés

**Affiliations:** 1 Microbial Ecophysiology Laboratory, CCTE Ciencia y Vida, Fundación Ciencia y Vida, Huechuraba 8580704, Chile; pedro.sepulveda.reb@gmail.com; 2 Centro de Bioinformática y Biología Integrativa, Facultad de Ciencias de la Vida, Universidad Andrés Bello, Santiago 8370146, Chile; 3 Centre for Ecology and Evolution in Microbial Model Systems, Linnaeus University, SE-391 82 Kalmar, Sweden; carola.mgr@gmail.com (C.G.-R.); mark.dopson@lnu.se (M.D.); 4 Instituto de Investigaciones en Matemáticas Aplicadas y en Sistemas, Unidad Académica del Estado de Yucatán, Universidad Nacional Autónoma de México, Mérida 97302, Yucatán, Mexico; ernesto.perez@iimas.unam.mx; 5 Center for Bioinformatics and Genome Biology, CCTE Ciencia y Vida, Santiago 7780272, Chile; 6 Facultad de Medicina y Ciencia, Universidad San Sebastián, Santiago 7510156, Chile

**Keywords:** sigma-factors, RpoN, motility, nitrogen metabolism, hydrogen, sulfur metabolism, enhancer-binding proteins, *Acidithiobacillia*

## Abstract

Extreme acidophiles from the *Acidithiobacillia* class thrive in highly acidic environments where they rely on diverse regulatory mechanisms for adaptation. These mechanisms include sigma factors, transcription factors (TFs), and transcription factor binding sites (TFBS), which control essential pathways. Comparative genomics and bioinformatics analyses identified sigma factors and TFs in *Acidithiobacillia*, showing similarities but key differences from reference neutrophiles. This study highlights sigma54-dependent one- and two-component systems that are crucial for survival in energy acquisition from sulfur compounds and hydrogen as well as nutrient assimilation. Furthermore, the data suggested evolutionary divergence in regulatory elements distinguishes S-oxidizing from Fe-S-oxidizing members of *Acidithiobacillia*. Conservation of gene clusters, synteny, and phylogenetic analyses supported the expected phenotypes in each species. Notable examples include HupR’s role in hydrogenase-2 oxidation in Fe-S-oxidizers, TspR/TspS regulation of the sulfur oxidation complex, and FleR/FleS control of flagellar motility in S-oxidizers. These regulatory mechanisms act as master controllers of bacterial activity, reflecting adaptation to distinct metabolic needs within *Acidithiobacillia*.

## 1. Introduction

Microorganisms that thrive in extremely acidic environments, characterized by low pH levels (below 3.0) and high metal ion concentrations [1,2,3], are vital catalysts for mineral oxidation. This process generates acidic solutions loaded with metals, commonly known as “acid rock drainage” and “acid mine drainage”. Their exceptional capabilities have led to various applications, including the biomining of metal sulfides, desulfurization of coal and natural gas, and numerous industrial processes [4,5,6]. Extreme acidophiles exhibit remarkable adaptability, adjusting their metabolism to tackle a wide range of challenges and environmental fluctuations, such as temperature variations, pH levels, electron acceptor/donor availability, CO_2_ concentrations, and nutrient deficiencies [4].

Over time, diverse acidophilic microorganisms have been isolated and classified, providing a wealth of genetic and phenotypic information from extremely acidic environments [7]. Within this spectrum, the chemolithotrophic extreme acidophilic bacteria from the *Acidithiobacillia* class [8] stand out, encompassing two primary families: the acidophilic *Acidithiobacillaceae* and the neutral-pH-adapted *Thermithiobacillaceae*. A recent reclassification of species within the genera distributed in *Acidithiobacillia* was introduced [9], and this reclassification has been further elaborated upon in subsequent studies [10,11]. These microorganisms exhibit a shared metabolic profile, relying on the oxidation of inorganic sulfur compounds for energy generation, known as thiotrophy [12]. Additionally, they autonomously convert CO_2_ into biomass by fixation processes [13]. Some species further display the capacity to oxidize ferrous iron for energy acquisition [14], while others are capable of performing hydrogen oxidation [15]. These phenotypes can be triggered as a response to environmental fluctuations such as energy or nutrient availability. Appropriate responses and metabolic switching strongly relies on transcriptional regulation, with a key role played by transcriptional regulatory elements including sigma factors (SFs), transcription factors (TFs), and their associated transcription factor binding sites (TFBS) [16].

Recently, a group of TFs was identified within the *Acidithiobacillia* class [11], contributing to extending our understanding of the transcriptional regulatory mechanisms in these extremophile bacteria and providing novel insights on the regulation of critical pathways necessary for their growth and survival in extremely acidic environments. However, there is little information regarding the distribution and function of SFs as master regulators of key physiological roles in response to environmental fluctuations.

**Figure 1:**
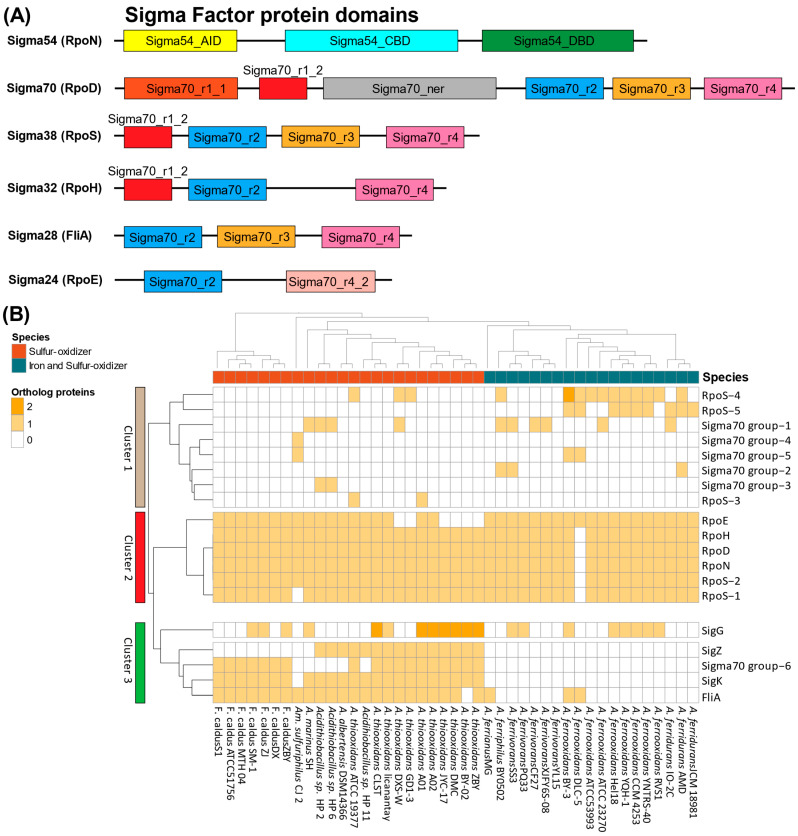
Phylogenetic distribution of *Acidithiobacillia* sigma factors. (**A**) Protein domain architecture of sigma54 and sigma70 proteins and (**B**) heatmap indicating presence/absence of known and potential SFs, based on protein orthology analysis. *Acidithiobacillia* representatives are phylogenetically arranged as represented by the dendrogram. Orthologous SFs counts are colored by the number (0–2) and rearranged in clusters according to Euclidean distance (left side dendrogram).

SFs are multi-domain protein subunits of bacterial RNA polymerase that play critical roles in transcription initiation, including the recognition and opening of promoters for the initial steps in RNA synthesis [17]. These proteins play an essential role in the regulation of specific genes that participate in several metabolic processes. SFs can be classified first by the major sigma70 family that includes the housekeeping sigma factor genes *rpoD* (sigma70) from group 1, and *rpoS* (sigma38), *rpoH* (sigma32), *fliA* (sigma28), and *rpoE* (sigma24) from groups 2–4 [17]. On the other hand, the sigma54 family includes the *rpoN* (sigma 54) [18,19]. Sigma54 proteins contain an activator interaction domain (Sigma54_AID, PF00309), a core binding region (Sigma54_CBD, PF04963), and a DNA-binding component (Sigma54_DBD, PF04552), defining a single differentiated protein family. In contrast, Sigma70 representatives in *Acidithiobacillia* are distributed into several groups [20,21] with Sigma70_r1_2 (PF00140) and Sigma70_r3 (PF04539) characteristic domains supporting the classification of groups 1–2 when both domains are present; these have been identified as RpoD, RpoS, and RpoH. Group 3 Sigma70 has a single Sigma70_r3 with high similarity to the classic FliA protein involved in flagellar expression. For a schematic representation of sigma factors domains architectures see Figure 1A. Group 4 (RpoE) shows only the presence of Sigma70_r2 and Sigma70_r4_2 domains. Domain architecture analysis strengthens the initial similarity-based identification and helps to define sigma factor diversity across *Acidithiobacillia* representatives. Unlike sigma70-mediated transcription initiation, sigma54-dependent regulation absolutely requires an activator to couple ATP hydrolysis energy to RNA polymerase–sigma54 complex isomerization for activation [22]. Sigma54 exhibits versatile characteristics, participating in diverse regulatory activities, which can respond to varied environmental signals [23].

SFs have been characterized in several model bacteria, including *Escherichia coli* [24], *Bacillus subtilis* [25], and *Clostridium* [26]. Their role has been linked to the regulation of diverse functions, including essential life processes and responses activated by specific stimuli, such as stress and morphological development [17,27]. Sigma factors also play a role in adaptive responses, such as sporulation, toxin production, and cell surface modifications [28].

SFs families can be recognized by their characteristic conserved binding site in the bacterial promoter. The sigma70 family contains the binding motif “TTGACA” from −35 and “TATAAT” from the −10 upstream promoter sequence [27]. In the case of the sigma54, conservation of “GC” from −24, and “TGC” from −12 were observed; additionally, this sigma factor strictly requires the ATP hydrolysis activity of an activating TF for the formation of open promoter complex [29]. These TFs are considered bacterial enhancer binding proteins (bEBPs) [22] and could work as one-component regulator, or two-component regulatory systems (TCSs). However, there is little information regarding the distribution and function of SFs as master regulators of key physiological roles in response to environmental fluctuations.

In *Acidithiobacillia*, SFs reports are restricted to gene expression analysis in the S-oxidizing ‘*Fervidacidithiobacillus caldus*’ (Formerly *Acidithiobacillus caldus*). These studies include investigations into the response to acid stress through RpoS regulatory activity [30] and functions related to flagellar biosynthesis and motility functions by FliA and RpoN [31].

TCSs trigger specific transcriptional responses of many important pathways, where a phosphotransfer reaction takes place between two conserved components: a histidine kinase sensor (HKS) that senses the stimulus, and a response regulator TF [32]. TFs, whose structure contains a DNA-binding domain (DBD), can recognize a transcription factor binding site motif (TFBS) in the promoter and/or regulatory regions. In addition, some TFs contain a sigma54 activating domain, which are part of the bEBPs families [22,33] and are able to work in a sigma54-dependent fashion, promoting coordinated responses of several pathways based one specific or several stimuli.

The transcriptional regulators NtrC, DctD, HydG, PilR, AlgB, HoxA, Hup1, RocR, and FlbD have been described as bEBPs [23]. bEBPs have been characterized in diverse bacterial models, including *Desulfovibrio vulgaris* [31], *E. coli* [34], *Salmonella typhimurium* [35], and *Sinorhizobium meliloti* [36]. Examples of these TCSs in *Acidithiobacillia* include the specific sigma54-dependent the TspS/TspR system, involved in the regulation of energy metabolism in ‘*F. caldus*’ MTH-04 [33], and the *Acidithiobacillus ferrooxidans* NtrB/NtrC pair, involved in nitrogen metabolism [37]. These TCSs have been shown to be essential for energy gathering and nutrient assimilation pathways in extreme acidophiles.

In this study, we present a comparative genomic exploration of 43 complete and draft genomes of *Acidithiobacillia* to detect SFs family proteins, their potentially associated TFs, and their regulatory targets. This comparative analysis was conducted to assess the function, distribution, and phylogeny of SFs and TFs to shed light on the roles of these systems in the *Acidithiobacillia* representatives, their association to speciation and the regulation of specific metabolic traits among clades. Furthermore, specific sigma54-dependent TCSs, their bEBPs, and their potential TFBSs have been also computationally identified. This provides an integrated view of the regulatory networks that might be responsible for specific physiological processes such as energy acquisition and nutrient assimilation key for survival and proliferation in highly acidic environments.

## 2. Materials and Methods

### 2.1. Genome Accessions

Complete and draft *Acidithiobacillia* genomes (*n* = 43) were obtained from NCBI for analysis (Table S1). CheckM (v1.2.2) [38] assessed the genome quality and completeness, requiring ≤10% contamination, ≥90% completeness, and >50% normalized compatible split length [39] (Table S2). Unannotated genomes underwent Prokka (v1.14.6) [40] prediction of coding regions and proteins with default parameters. EggNOG (v2.1.9) [41] and InterProScan (v5.55-88.0) [42] then assigned functional annotation and classification using default parameters.

### 2.2. Phylogenetic Analysis

A phylogenomic tree was then constructed to corroborate the *Acidithiobacillia* taxonomy. Concatenated alignments were generated from core conserved proteins extracted from the 43 genomes using MAFFT (v7.407) with L-INS-I iterative refinement [43]. GBLOCKS (v0.91b) [44] trimmed unreliable alignment regions before concatenation. Maximum likelihood trees were then built by IQtree (v 1.6.12) [45] using the concatenated refined alignments, with 1000 bootstraps for robustness, and a substitution model from IQtree [46]. The final tree was visualized in Figtree (http://tree.bio.ed.ac.uk/software/figtree/; accessed on 1 May 2023).

### 2.3. Orthology and Protein Family Analysis

Comparative genomics focused on protein orthologous groups (OGs) across *Acidithiobacillia* representatives. OGs were defined using ProteinOrtho (V6.0.22) [47] with bidirectional best BLASTp hits between genomes. The criteria were: BLASTp E-value under 10^−10^, over 60% identity, 60% query coverage, and other default parameters. Each OG then underwent HMMER3 (v3.4) [48] hidden Markov model searches against Pfam- database v33.1 [49] for protein family classification.

### 2.4. Identification of Genes and Pathways Across Acidithiobacillia Genomes

The metabolic reconstruction analysis involved selecting curated gene- and protein-coding sequences from species with available experimental data. Literature mining and manual curation identified genes encoding proteins crucial for processes such as sulfur oxidation, nitrogen assimilation/reduction, and hydrogen uptake/evolution (Table S3).

### 2.5. Recovery of Transcriptional Units and Upstream Regions

Gene clusters and potential transcriptional units (TUs) were identified using available experimental data from sources including [7,50,51,52,53,54] and the AciDB database (v1.0) [55]. Additionally, putative gene clusters and TUs were inferred based on intergenic distances between neighboring genes, following established bacterial protocols. In this context, an “upstream region” was defined as having more than 40 bases between coding sequences.

### 2.6. Identification and Phylogeny of Sigma Factors in Acidithiobacillia Representatives

Sigma factors were identified by comparative genomics using experimentally validated SFs from the model species *E. coli* K-12 (from RegulonDB; https://regulondb.ccg.unam.mx/; accessed on 1 May 2022) and *B. subtilis* 168 (from DBTBS; https://dbtbs.hgc.jp/; accessed on 1 May 2022). Additionally, gene annotations, descriptions, and Pfam domains were data-mined to find further Sigma70-like proteins. Phylogenetic trees for sigma70 and sigma54 families were built using previous protocols, with validated SF sequences from *E. coli* K-12 and *B. subtilis* 168 as outgroup references. Sigma54 and sigma70 protein family orthologs were assessed via earlier orthology analysis. Heatmaps showing SF ortholog groups diversity, quantity, and distribution were generated in Rstudio using the pheatmap v1.0.12 package. *Acidithiobacillia* representatives were clustered according to their phylogeny, and ortholog SF groups were clustered by Euclidean distance.

### 2.7. Identification of Sigma54-Dependent Two-Component Systems

Identification of bEBPs that could be participating in TCSs was implemented by searching Pfam domains to select proteins with the sigma54 activator (PF00158.27) and response regulator (PF00072.25) domains. The HKS proteins were identified by a curated search examining the genomic architecture of previously recovered transcriptional units and locating those containing both HKS and bEBP genes. Additionally, the signal transduction histidine kinase domain (PF00512) Pfam annotation was identified in HKS proteins.

### 2.8. Identification and Analysis of Putative DNA Binding Sites

A motif footprint approach was used in MEME (Multiple Em for Motif Elicitation, which detects novel conserved motifs in input sequences) (v 5.4.1) to identify potential DNA binding sites [56] while focusing on upstream regions of studied transcription units containing known genes. The MEME parameters were: up to 10 motifs, the ZOOPS algorithm to allow zero or one site per sequence, a 4th-order Markov background model to normalize biases, and motif lengths from 5 to 20 nucleotides. Sequence logos were also generated using WebLogo (v 2.8.2) [57] to visualize sequence conservation and relative nucleic acid frequencies at each position for the predicted motifs from transcription factors and sigma factors by applying the Schneider and Stephens method [58].

### 2.9. Matrix Scanning of Binding Sites

Potential DNA binding sites were identified using Position Weight Matrices (PWMs) in 15 *Acidithiobacillia* reference genomes along with experimentally validated *E. coli* K-12 binding site sequences for bacterial transcription factors and sigma factors from RegulonDB. “sites2meme” (from MEME suite) was then used to construct PWMs from these known binding sites. Fourth-order Markov background models were built for *E. coli* K-12 and the 15 reference species using the “create-background-model” tool from the RSAT suite (v2018) [59]. The FIMO tool then scanned (with default parameters) the PWMs against upstream regions of transcription units, using matched background models. Retrieved sites were filtered based on *p*-value thresholds from RegulonDB or a default *p*-value under 1 × 10^−4^. Overlapping sites were removed, keeping those over 50% sequence coverage with the best score and the remaining sites were restricted according to the selected reference genes. Sequence logos summarize these putative binding sites.

## 3. Results

### 3.1. Acidithiobacillia Display a Diverse Sigma Factor Repertoire Providing Clues on Extreme Acidophile Adaptability

*Acidithiobacillia* representatives were organized into the five major clades proposed by Moya-Beltrán et al. (2021) [9], where three phylogroups were identified as mesophilic sulfur-oxidizers (S-oxidizers), thermotolerant S-oxidizers, and mesophile/eurypsychrophilic iron–sulfur oxidizers (Fe-S-oxidizers) (Figure S1).

Identification of *Acidithiobacillia* SFs using the complete repertoire from the *E. coli* protein models (RpoN, RpoD, RpoS, RpoH, FliA, RpoE), a partial group from *B. subtilis* (SigK, SigZ, SigG), and proteins containing sigma70 conserved domains from the Pfam database is presented in Figure 1. A schematic representation of these domain architectures is shown in Figure 1A, using ‘*F. caldus*’ ATCC 51756 plus *Acidithiobacillus thiooxidans* ATCC 19377 for S-oxidizers and *A. ferrooxidans* ATCC 23270 for Fe-S-oxidizers as representative examples.

An orthology-based SFs distribution analysis identified three main clusters in the *Acidithiobacillia* (Figure 1B). Cluster 1 included five sigma70-like proteins and three orthologous groups of RpoS (RpoS_3, RpoS_4, and RpoS_5). Notably, RpoS_4 and RpoS_5 exhibited a predominant distribution among Fe-S-oxidizers in comparison to other clades. In cluster 2, a core of conserved groups of sigma factors was identified, encompassing five out of the six sigma factor groups present in *E. coli* (RpoD, RpoN, RpoE, RpoH, and two RpoS groups). This cluster was present in nearly all analyzed genomes, except for *A. ferrooxidans* DLC-5, notably characterized by its fragmented assembly. Within cluster 3, the FliA sigma factor was identified alongside with SigK, SigZ, and sigma70-like group 6, showing a predominant presence among S-oxidizers. Notably, SigG was widely distributed across almost all taxonomic groups considered in the analysis, showing differences in gene dosage in *A. thiooxidans* and the complete absence of SigZ in ‘*F. caldus*’ strains.

### 3.2. Sigma Factors Phylogeny Reflects Divergence Signatures of Acidithiobacillia Phenotypes

**Figure 2:**
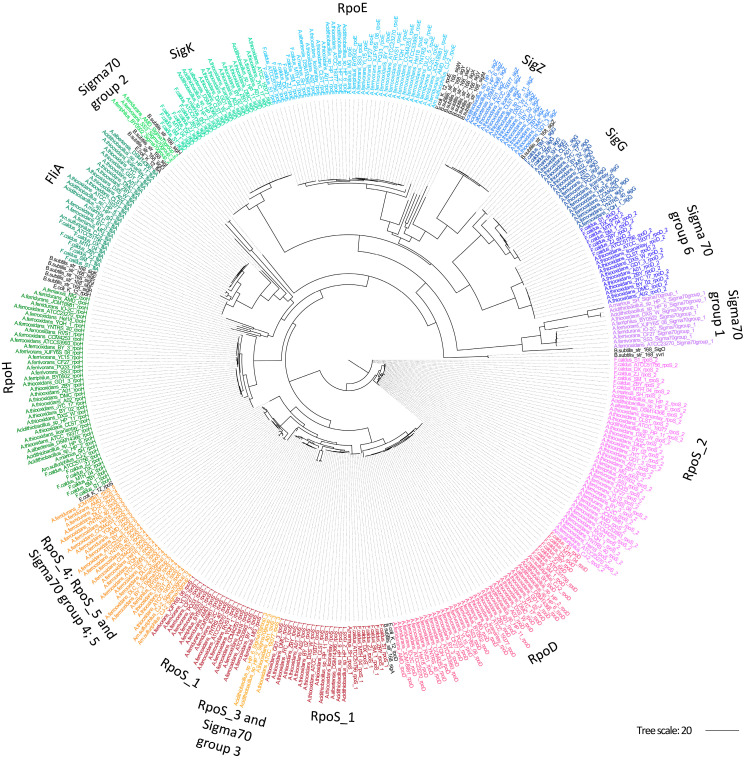
Phylogenetic relationships between sigma70 family proteins in *Acidithiobacillia*. Maximum-likelihood phylogenetic tree based on the sigma70 proteins identified in *Acidithiobacillia*. Groups of SFs were colored by species. Model *E. coli*, and *B. subtilis* representative SFs are shown in black.

To assess the phylogenetic relationships between *Acidithiobacillia* Sigma70 candidates, a maximum-likelihood phylogeny was generated using all potential SFs proteins for the two primary families, sigma70 and sigma54. The generated sigma70 phylogeny (Figure 2) revealed several clades containing individual groups of SFs, including RpoD, RpoE, RpoH, RpoS_2, FliA, SigG, SigK, and SigZ, along with sigma70-like groups (groups 1, 2, and 6). However, there was an exception of one sub-clade containing multiple protein groups including RpoS (RpoS_1, RpoS_3, RpoS_4, and RpoS_5) and sigma70-like groups (groups 3, 4, and 5). RpoS_2 was found to be distributed in an independent branch distantly separated from the other RpoS groups. Selected outgroups in this analysis included SFs from *E. coli* and *B. subtilis* (Figure 2, depicted in black) that show a distinctive position typically outside the *Acidithiobacilli* branches. As expected from their taxonomical origin, *B. subtilis* SFs were more distantly related than *E. coli* representatives.

**Figure 3:**
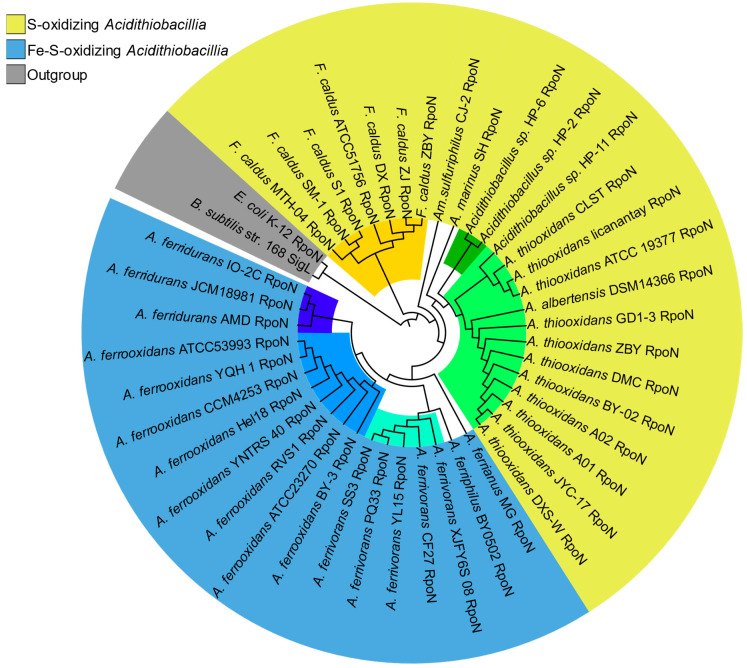
Phylogeny of *Acidithiobacillia* sigma54 orthologs. Maximum-likelihood phylogenetic tree based on *Acidithiobacillia* sigma54 (RpoN) representatives. Sigma54s are colored by phenotype, represented by outer layout color, and species lineages are represented by inner sub-clade colors (colored tree branches). Model species outgroups (*E. coli* and *B. subtilis*) are highlighted in gray.

As expected, the sigma54 phylogeny (Figure 3), characterized by a unique RpoN protein family, showed high concordance with the species’ phylogenetic distribution supporting the vertical inheritance of this sigma factor.

### 3.3. Sigma54: A Master Regulator for Energy and Nutrient Assimilation in Acidithiobacillia?

**Figure 4:**
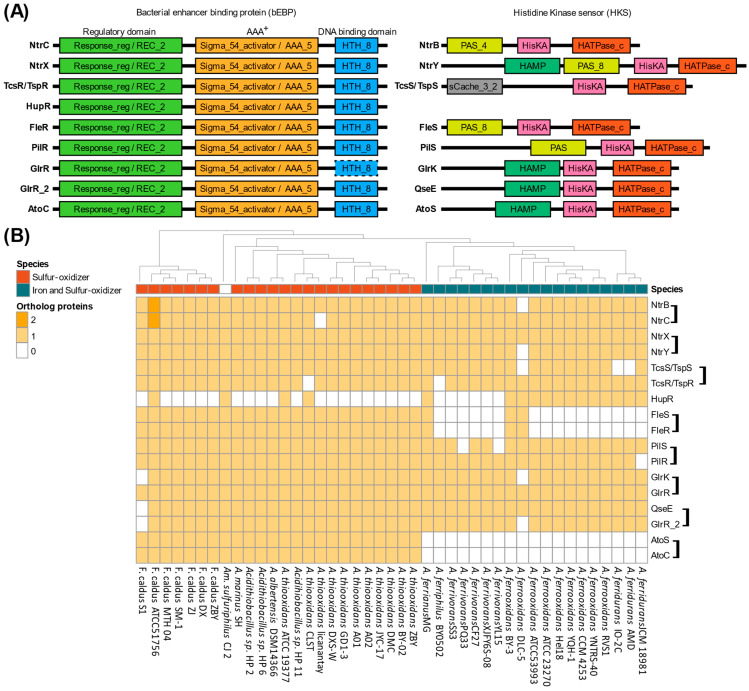
Sigma54-dependent two-component systems repertoire in *Acidithiobacillia*. (**A**) Protein domain architecture of sigma54-dependent TCSs separated by response regulator bEBP component and histidine kinase sensor component. AAA^+^, ATPase associated with cellular activities; REC, phosphoacceptor receiver domain; HTH, helix–turn–helix domain; PAS, Per–Arnt–Sim domain; HisKa, Signal transduction histidine kinase; HATPase_c, histidine kinase/HSP90-like ATPase. A dashed frame denotes that the HTH_8 domain was not detected in GlrR with the applied parameters, but its presence is inferred from its identification in the GlrR_2 group. (**B**) Protein ortholog groups in *Acidithiobacillia* representatives with TCSs grouped in a bracket with the histidine kinase sensor first, followed by the transcription factor.

To elucidate Sigma54’s influence across key metabolic pathways, a protein domain prediction approach was implemented based on Sigma54 activator (PF00158.27) and response regulator (PF00072.25) domain searches across the *Acidithiobacillia* representatives. A total of 546 proteins were identified containing a Sigma54 activator domain and 617 with a response regulator domain (Table S4, Supplementary Data). Of these, 318 proteins contained both domains, constituting the identified bEBPs from one-component and TCS (bEBP and HKS). These proteins were distributed across nine OGs, including HupR, NtrC/NtrB, NtrX/NtrY, TspR/TspS (or TcsR/TcsS), FleR/FleS, PilR/PilS, GlrR/GlrK, GlrR_2/QseE, and AtoC/AtoS (Figure 4). The TCSs comparison using referential representatives consistently revealed three-domains for the bEBP response regulator component, including the response regulator (also called as REC_2), sigma54 activator (AAA^+^), and the DNA-binding (HTH_8) domains [22]. However, some variations were observed in HKS proteins containing cytoplasmic domains involved in signal input/transmission (PAS domain) or signal transduction domains (HAMP domain). In addition, TspS showed an additional domain, a single cache domain 3 (sCache_3_2), described within the extracellular sensors in bacteria [60]. Nonetheless, all the HKS found in *Acidithiobacillia* contain the conserved domains phosphoacceptor Histidine Kinase A (HisKA) and the catalytic domain HATPase_c (Figure 4A) that are essential for TCS functions. These conserved orthologous genes highlighted conserved systems in all *Acidithiobacillia* (Figure 4B), except for AtoS/AtoC and FleS/FleR exclusive to most S-oxidizers. However, the HKS was absent for the HupR/HupT hydrogen oxidation TCS in all *Acidithiobacillia*, with only the HupR bEBP one-component regulator conserved.

**Figure 5:**
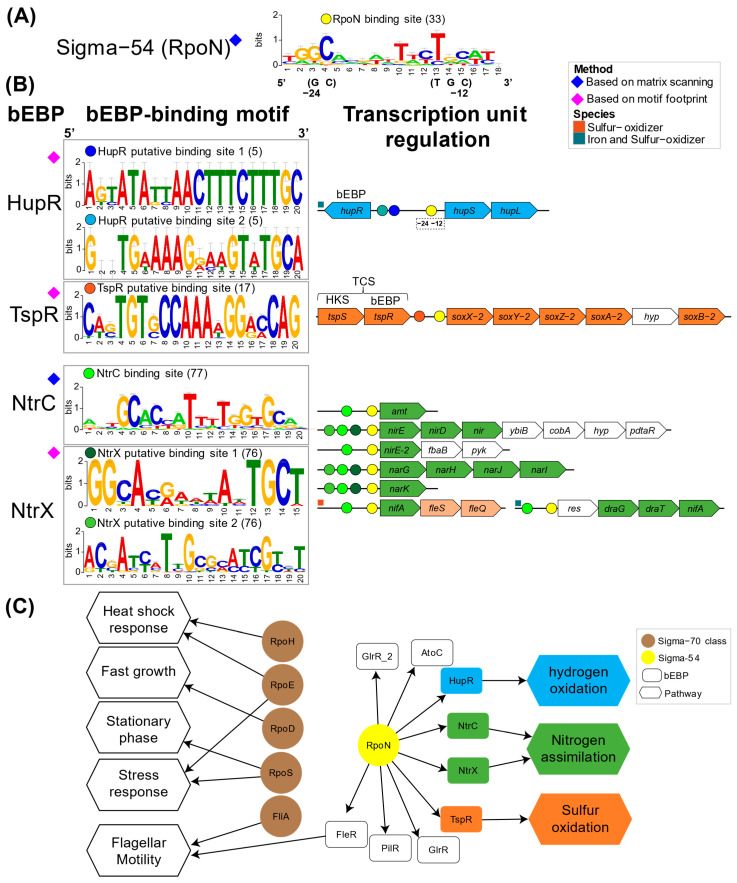
Transcriptional regulatory circuits of bacterial enhancer binding proteins in *Acidithiobacillia*. (**A**) RpoN binding site and (**B**) bEBP binding sites and regulatory activity retrieved for known and de novo inferences. The number of binding sites that build the logos graph are given in parentheses. Transcriptional units are represented by referential species, where bEBP binding sites are depicted in color circles in the promoter regions. Genes encoding metabolic pathways of hydrogen in cyan, nitrogen in green, sulfur in orange, and additional genes in white; *res*, response regulator protein. (**C**) Transcriptional regulatory network representation of sigma factors and bEBPs.

The identification of putative DNA binding sites, the genes and operon potentially under control, cognate transcription, and sigma factors enabled the reconstruction of transcriptional regulatory circuit (Figure 5). This reconstruction was centered on curated and conserved orthologous genes across the *Acidithiobacillia* (Figure S2) with reported bEBP-controlled operons or suggested pathway-linked regulation. Information from binding site prediction from sigma54 (RpoN) (Figure 5A) and key bEBPs (Figure 5B) were generated allowing the identification of regulators for hydrogen (HupR), nitrogen (NtrC and NtrX), and sulfur (TspS) pathways. Experimental information from validated sites from other bacteria enabled recognition of likely regulated targets via PWM scanning, such as RpoN and NtrC. On the other hand, for non-reported binding sites (without known PWM) from bEBP HupR, TspR, and NtrX, the utilization of a motif footprint approach pinpointed conserved upstream binding motifs in suggested pathway-linked regulation genes. Referential *Acidtihobacillia* species were utilized to represent operon organization (Figure 5B, right-side). HupR and TspR co-localized with the suggested genes they regulate (*hupS-hupL*), whereas the remaining studied bEBPs were located in separate genomic regions, distinct from their target genes. The NtrC binding site scanning also enabled proposals for regulatory interactions in diverse transcriptional units. Bringing these together allowed the reconstruction of an NtrC regulon centered on nitrogen pathways in the *Acidithiobacillia*. Since NtrX shares related regulatory activities, participation in similar regulatory interactions as NtrC can be inferred. A summarized regulatory network captures sigma factor master regulator connectivity as well as bEBP regulatory roles across examined metabolic pathways (Figure 5C).

## 4. Discussion

### 4.1. An Acidophilic Lifestyle Demands Robust Gene Regulation Circuitry

While sigma70 family regulatory proteins participate globally in all essential bacterial processes, sigma54 has been demonstrated as an indispensable master regulator specifically controlling vital pathways [18]. The *Acidithiobacillia* SFs (Figure 1A) revealed the presence of identical domains as observed in the sigma factor family classification [20,61]. This suggested adaptations to regulate essential processes enabling acidophile survival and growth in extremely acidic settings. However, additional proteins with sigma54-associated domains were identified that lacked a defined annotation and without comparison in other bacterial models. Almost the entire set of *E. coli* SFs was conserved in the *Acidithiobacillia* class except for FliA, which was conserved mainly in S-oxidizers (Figure 1B). FliA controls the expression of flagella-related genes [62]. Interestingly, the S-oxidizing ‘*F. caldus’* and *A. thiooxidans* share the gene potential and capacity to develop flagellum and chemotaxis [30,63,64,65]. Therefore, it was suggested that FliA may also participate in regulating flagellar genes. In addition, FliA was identified in some Fe-S-oxidizers, such as *A. ferrooxidans* strains where genes involved in chemotaxis have been previously identified [65,66].

### 4.2. Sigma Factor Master Regulators Tend to Mirror Acidithiobacillia Evolution

The sigma70 phylogeny showed most groups forming individual sub-clades near reference outgroups. Sigma70-like group 6 displayed just one domain shared by RpoE (Table S5, Supplementary Data) and had the shortest protein sequence, suggesting a new group of sigma factors with intriguing regulatory activity in acidophiles yet to be determined. Additionally, we find RpoS instances. One sub-clade contained four RpoS and three sigma70-like groups, indicating these latter may associate with RpoS. Furthermore, the deep-branching RpoS_2 cluster differed from the reference outgroups and lacked one conserved RpoS domain (Table S5, supplementary material), distinguishing it from other RpoS proteins. RpoS_2 may have emerged within the class *Acidithiobacillia* or been acquired through horizontal gene transfer from another member of the acidophilic community, highlighting the dynamic genetic exchanges that shape adaptation to extreme acidic environments and its differentiation from neutrophilic species described.

The sigma54 (RpoN) phylogeny (Figure 3) mirrored the taxonomic structure of the broader *Acidithiobacillia* phylogenomic tree. This agreement provides evidence cementing *rpoN* as a fundamental housekeeping gene perpetuating acidophilic viability across the taxon.

In the case of SigZ, originally identified in *B. subtillis*, it constituted the main difference in the repertoires of ‘*F. caldus*’ strains in comparison to mesophilic sulfur-oxidizers like *A. thiooxidans*, which clustered separately despite their phenotypic similarities. While ‘*F. caldus*’ shared a similar sulfur oxidation profile and many sigma factor orthologs with mesophilic sulfur-oxidizers such as *A. thiooxidans*, the separation into a distinct cluster was likely explained by lineage-specific gene differences due to their gene dosage adaptations, potentially driven by moderately thermophilic conditions.

### 4.3. Sigma54-Dependent Two-Component Systems in Acidithiobacillia Were Potentially Linked to Key Metabolic Pathways

The discovery of *Acidithiobacillia* sigma54-dependent two-component systems that were involved in key metabolic pathways advances understanding of their dominance in extremely acidic environments [67]. Sulfur oxidation TCSs regulate protein complexes that enable the utilization of diverse reduced inorganic sulfur compounds for energy acquisition [52]. In ‘*F. caldus*’, TspS/TspR is co-located with the *sox* sulfur oxidation complex gene cluster [33,68], mainly conserved in S-oxidizing representatives and notably absent in other Fe-S-oxidizers except for *A. ferrivorans*, which shows a partial conservation of this gene cluster [9]. TspS/TspR’s DNA-binding site and Sigma54 promoter interaction suggest transcriptional regulation of *sox* genes in S-oxidizers [11]. Furthermore, the TcsS/TcsR system described in Fe-S-oxidizers and localized adjacent to the *tetH-doxDA* gene cluster [69] was orthologous to TspS/TspR, (Figure 4B) and conserved identical domains Their specialized regulatory roles were likely adapted to control a phenotype-specific metabolism in S-oxidizers and Fe-S-oxidizers.

Hydrogen oxidation also supplies energy, a process carried out by different protein complexes categorized into hydrogenase groups [70]. The one-component HupR system was situated in a divergent orientation relative to hydrogenase group 2 (*hupS*-*hupL*) and primarily conserved in Fe-S-oxidizers. While HupR has been suggested to function as part of a two-component regulatory system, it may also exert its regulatory activity independently, without requiring phosphorylation by a histidine kinase [71], suggesting a versatile role in hydrogenase regulation.

Nitrogen scarcity in extremely acidic environments is offset by the nitrogen fixation capability of some *Acidithiobacillia* [72] to produce bioavailable nitrogen compounds, supporting the broader microbial communities in these environments. The NtrB/NtrC and NtrX/NtrY TCSs regulate some of these nitrogen assimilation pathways genes [73] while NtrB/NtrC is described in other microorganisms [74] such as *Rhodobacter capsulatus* [75], *A. ferrooxidans* DSM 16786, and *A. thiooxidans* DSM 17318 [76]. NtrB/NtrC senses nitrogen sources like molecular nitrogen, ammonia, and nitrate, and it was suggested to control nitrogen assimilation in most *Acidithiobacillia* due to their complete conservation (Figure 4). Assimilation mechanisms for nitrate (*nar*) and nitrite (*nir*) are present in some *Acidithiobacillia* [76] (Figure S2). Therefore, NtrB/NtrC may regulate these compounds’ utilization by modulating related gene transcription. Though *ntrB-ntrC* clusters individually within genomes, there were no nearby genes associated with nitrogen metabolism. Additionally, the TCS NtrX/NtrY detects ammonia levels, demonstrated in the diazotrophs such as *Azospirillum brasilense* [77], and identified in the acidophilic *Leptospirillum ferriphilum* DSM 17947 [76]. NtrX/NtrY appeared widely in *Acidithiobacillia* (Figure 4), suggesting NtrY-mediated ammonia sensing and NtrX regulation. The *ntrX-ntrY* genes fell within a five or six gene operon across *Acidithiobacillia*, also lacking nearby nitrogen metabolism genes. Notably in *A. ferrooxidans* ATCC 23270, NtrX/NtrY was nearby to TcsS/TcsR. NtrC/NtrB and NtrX/NtrY TCSs share protein domains with those in *Herbaspirillum seropedicae* [73]. Thus, this supported the nitrate and nitrite regulatory activity via these TCS components found in *Acidithiobacillia*.

Flagellar motility enables chemotaxis and environmental signaling response in bacteria [78]. Flagellar regulation involves transcriptional cascades controlled by the bacterial enhancer-binding protein FleQ, demonstrated in organisms like *Pseudomonas aeruginosa* [79] and *Legionella pneumophila* [80]. Flagellar motility uniquely occurs in S-oxidizing *Acidithiobacillia*, which conserves the Class II flagellar genes [30]. These likely fall under regulation by RpoN, FleQ, FleS/FleR, and FliA [30]. Notably, the sigma54-dependent *fleS/fleR* genes were collocated with *fleQ*, suggesting a key conserved regulatory gene cluster. The bEBP FleR was proposed to control the transcription of the Class III flagellar rod encoding genes (*flgBCDEFGHIJKL*), which encode the flagellar basal body rod [30].

## 5. Conclusions

This work unveils previously uncharacterized regulatory circuits that may govern the dominance of extreme acidophiles in natural and anthropogenic extreme acidic environments. Sigma factors (SFs) and bacterial enhancer-binding proteins (bEBPs) orchestrate the regulation of key pathways involved in hydrogen, nitrogen, and sulfur metabolism in the *Acidithiobacillia* class. These regulatory tools not only empower acidophiles to exploit metals and nutrients in industrial settings, but also likely played a role in early metabolic evolution and continue to shape contemporary acidophilic microbial communities via biogeochemical cycles. Through the comprehensive identification of SF family proteins and their associated TFs across 43 *Acidithiobacillia* genomes, this study offers insights into their distribution, evolutionary patterns, and potential involvement in speciation and clade-specific metabolic traits. The computational detection of sigma54-dependent TCSs, their bEBPs, and potential TFBSs suggested a coordinated regulatory framework governing essential metabolic functions. These elements are likely critical for energy acquisition and nutrient assimilation, underpinning survival and proliferation in extremely acidic environments. By revealing complex yet adaptable transcriptional regulatory networks, this research not only highlights a sophisticated acid life strategy but also lays the groundwork for future experimental validation of these computational predictions, aiming to confirm their physiological significance in *Acidithiobacillia*.

## Data Availability

The datasets used in this study were obtained from public databases hosted by NCBI and are available in online repositories.

## Supplementary Materials

The following supporting information can be downloaded at: https://www.mdpi.com/article/10.3390/microorganisms13061199/s1, Table S1: Properties of 43 *Acidithiobacillia* assemblies; Table S2: CheckM assessment results in *Acidithiobacillus* genus; Table S3: List of selected genes coding sequences involved in metabolic pathways; Figure S1: Phylogeny and reported phenotypes of 43 strains of *Acidithiobacillia* representatives; Figure S2: Phylogenetic distribution of selected proteins ortholog groups involved in assessed metabolic pathways in *Acidithiobacillia*. Supplementary material (Excel file), Table S4: Identification of Sigma54 activators and Response regulators across all strains. Each regulator type is presented in a separate section, side by side. Table S5: Domain and Family identification of Sigma Factor proteins

## Abbreviations

The following abbreviations are used in this manuscript:

TF	transcription factor
TFBS	transcription factor binding site
TCS	two-component system
SF	sigma factor
bEBP	bacterial enhancer binding proteins
HKS	histidine kinase sensor
DBD	DNA-binding domain
OG	orthologous group
TU	transcriptional unit
PWM	Position Weight Matrix
