# Supplementary material for: Comparative Genomics of Sigma Factors in Acidithiobacillia Sheds Light into the Transcriptional Regulatory Networks Involved in Biogeochemical Dynamics in Extreme Acidic Environments
Source: Microorganisms. 2025 May 24;13(6):1199. doi: 10.3390/microorganisms13061199 (PMC12194929; doi:10.3390/microorganisms13061199)
Supplement: Supplementary file 1 [file microorganisms-13-01199-s001.zip › supplementary files.pdf]

Table S1. Properties of 43 *Acidithiobacillia* assemblies used in this study.

Feature	NCBI Accession[1]	State[2]	Size (Mb)	#Chr/Scaffold	#Total Protein	Geographical Origin
**Mesophilic sulfur-oxidizers**						
***Acidithiobacillus albertensis* DSM 14366** [A]	GCA_001931655.1	D	3.50332	0/141	3592	Canada: Alberta
***Acidithiobacillus thiooxidans* A01** [A]	GCA_000559045.1	D	3.82016	0/213	4029	China
***Acidithiobacillus thiooxidans* A02**	GCA_001705645.1	D	3.72088	0/351	3627	China: Jiangxi, Pingxiang
***Acidithiobacillus thiooxidans* ATCC 19377**	GCA_009662475.1	C	3.415726	1/0	3251	China
***Acidithiobacillus thiooxidans* BY-02**	GCA_001705725.1	D	3.81297	0/449	3688	China: Gansu, Baiyin
***Acidithiobacillus thiooxidans* CLST** [A]	GCA_002079865.1	D	3.97495	0/40	4147	Chile: Salar de Gorbea, Atacama Region
***Acidithiobacillus thiooxidans* DMC**	GCA_001705625.1	D	3.85414	0/270	3763	China: Hunan, Chenzhou
***Acidithiobacillus thiooxidans* DXS-W**	GCA_001705805.1	D	3.94685	0/319	3810	China: Xinjiang, Hami, Dongxiang Mountain
***Acidithiobacillus thiooxidans* GD1-3**	GCA_001705695.1	D	3.94694	0/335	3809	China: Guangdong, Shaoguan
***Acidithiobacillus thiooxidans* JYC-17**	GCA_001705755.1	D	3.8317	0/371	3735	China: Gansu, Baiyin
***Acidithiobacillus thiooxidans* ZBY**	GCA_001756595.1	D	3.79342	0/277	3712	Zambia: Chambishi
***Acidithiobacillus thiooxidans* licanantay** [A]	GCA_000709715.1	D	3.9379	0/345	4263	Chile: Atacama
***Acidithiobacillus sp.* HP-11**	GCA_015100075.1	D	2.954837	0/113	2885	USA: New York
**Thermotolerant sulfur-oxidizers**						
***Fervidacidithiobacillus caldus* ATCC 51756** [R P]	GCA_000175575.2	C	2.98705	4/0	2933	England, North Warwickshire

Fervidacidithiobacillus caldus **DX**	GCA_001756675.1	D	3.12221	0/390	2841	China: Jiangxi
Fervidacidithiobacillus caldus **MTH-04** [P]	GCA_001650235.2	C	2.97576	3/0	2713	China: Tenchong area, Yunnan province
Fervidacidithiobacillus caldus **S1**	GCA_001756775.1	D	2.79279	0/1208	2396	China: Jiangxi
Fervidacidithiobacillus caldus **SM-1** [P]	GCA_000221025.1	C	3.2376	5/0	3186	-
Fervidacidithiobacillus caldus **ZBY**	GCA_001756725.1	D	3.16007	0/414	2873	Zambia: Chambishi
Fervidacidithiobacillus caldus **ZJ**	GCA_001756745.1	D	3.14308	0/386	2864	China: Fujian
**Mesophile/eurypsychrophilic iron- and sulfur-oxidizers**						
Acidithiobacillus ferrianus **MG**	GCA_010378095.1	D	3.166	0/90	3020	Greece:Milos
Acidithiobacillus ferridurans **AMD**	GCA_008926505.1	C	2.933811	1/0	2833	Canada: Sudbury
Acidithiobacillus ferridurans IO-2C	GCA_003309025.1	D	2.71689	0/23	2634	USA: Texas
Acidithiobacillus ferridurans **JCM 18981**	GCA_003966655.1	C	2.9214	1/0	3026	Japan:Okayama
Acidithiobacillus ferriphilus BY0502	GCA_001652185.1	D	2.97667	0/295	2816	China: Gansu
Acidithiobacillus ferrivorans **CF27** [P]	GCA_900174455.1	C	3.45611	2/0	3867	-
Acidithiobacillus ferrivorans **PQ33** [P]	GCA_001857665.2	D	3.32048	0/103	3063	Peru: Pasco
Acidithiobacillus ferrivorans **SS3**	GCA_000214095.3	C	3.20755	1/0	3093	Norilsk, Russia
Acidithiobacillus ferrivorans **XJFY6S-08**	GCA_016250455.1	C	3.16138	1/0	2960	China:xinjiang
Acidithiobacillus ferrivorans **YL15**	GCA_001685225.1	D	2.99658	0/190	2795	China: Tibet

***Acidithiobacillus ferrooxidans* ATCC 23270**[R]	GCA_000021485.1	C	2.9824	1/0	3147	-
***Acidithiobacillus ferrooxidans* ATCC 53993**	GCA_000020825.1	C	2.88504	1/0	2826	-
***Acidithiobacillusferrooxidans* BY-3**[A]	GCA_010577825.1	D	3.832339	0/194	4061	China: Baiyin, Gansu
***Acidithiobacillus ferrooxidans* CCM 4253**	GCA_003233765.1	D	3.19656	0/15	3059	Czech Republic: Zlate Hory
***Acidithiobacillus ferrooxidans* DLC-5**[A]	GCA_000732185.1	D	4.18422	0/2090	4696	China: Heihe, Heilongjiang, Wudalianchi
***Acidithiobacillus ferrooxidans* Hel18**[A]	GCA_001559335.1	D	3.10916	0/123	3180	TU Bergaakademie Freiberg
***Acidithiobacillus ferrooxidans* RVS1**	GCA_003931975.1	D	2.82631	0/49	2705	Argentina:Neuquen
***Acidithiobacillus ferrooxidans* YNTRS-40**[P]	GCA_013462805.1	C	3.257037	2/0	3078	-
***Acidithiobacillus ferrooxidans* YQH-1**[A]	GCA_001418795.1	D	3.11122	0/96	2956	China
**Not reported**						
***Am. sulfuriphilus* CJ-2**	GCA_003721225.1	D	2.81686	0/195	2738	United Kingdom:Gwynedd
***Acidithiobacillus marinus* SH**	GCA_002847505.1	D	2.90361	0/65	2844	Japan: Okayama, Seto-Inland Sea
***Acidithiobacillus* sp. HP-2**	GCA_015100155.1	D	3.200419	0/48	3111	USA: New York
***Acidithiobacillus* sp. HP-6**	GCA_015100135.1	D	3.209818	0/52	3109	USA: New York

[1] NCBI Genbank assembly accession ID. [2] Sequence state: C: complete sequenced genome, D: draft sequenced genome. Chr: chromosome. [R] Reference strain for species; [A] reannotated in this study; [P] Assemblie with plasmids.

Table S2: CheckM assessment results in *Acidithiobacillus* genus. Consideting Completeness(Cp) > 90; Contamination(Ct) < 10; Normalization (CP-5(Ct)) > 50 (Parks et al., 2015; Parks et al., 2017).

***Acidithiobacillus* strain**	**Completeness**	**Contamination**	**Normalization**
F. caldus ZBY	99.38	2.8	85.38
F. caldus SM-1	99.38	2.17	88.53
F. caldus MTH-04	99.38	2.02	89.28
F. caldus DX	99.38	2.8	85.38
F. caldus ATCC 51756	99.38	1.55	91.63
A. marinus SH	99.34	0.93	94.69
A. thiooxidans licanantay	99.34	2.24	88.14
A. thiooxidans ZBY	99.34	1.86	90.04
A. thiooxidans JYC-17	99.34	1.86	90.04
A. thiooxidans DXS-W	99.34	1.24	93.14
A. thiooxidans DMC	99.34	1.86	90.04
A. thiooxidans ATCC 19377	99.34	1.24	93.14
A. thiooxidans GD1-3	99.34	0.93	94.69
A. thiooxidans A02	99.34	1.86	90.04
A. ferrooxidans YQH-1	99.34	0.03	99.19
A. ferooxidans YNRTS-40	99.34	1.86	90.04
A. ferrooxidans RVS1	99.34	0	99.34
A. ferrooxidans IO-2C	99.34	0	99.34
A. ferrooxidans Hel18	99.34	0	99.34
A. ferrooxidans CCM 4253	99.34	0	99.34
A. ferrooxidans ATCC 53993	99.34	0	99.34

A. ferrooxidans ATCC 23270	99.34	0	99.34
A. ferrivorans SS3	99.34	1.86	90.04
A. ferrivorans PQ33	99.34	2.64	86.14
A. ferrivorans CF27	99.34	2.33	87.69
A. ferridurans JCM18981	99.34	0	99.34
A. albertensis DSM 14366	99.23	0.62	96.13
A. thiooxidans BY-02	99.03	1.86	89.73
A. ferrivorans YL15	99.03	1.37	92.18
A. thiooxidans A01	98.72	1.24	92.52
A. ferriphilus BY0502	98.25	2.59	85.3
F. caldus ZJ	98.14	4.37	76.29
Am. sulfuriphilus CJ-2	98.06	0.67	94.71
A. thiooxidans CLST	97.77	1.24	91.57
A. ferrooxidans DLC-5	95.4	5.06	70.1
F. caldus S1	90.37	3.18	74.47
A. sp. HP-6	99.34	0.62	96,24
A. sp. HP-2	99.34	0.62	96,24
A. sp. HP-11	99.34	0.62	96,24
A. ferrooxidans BY3	99.34	1.24	93,14
A. ferrivorans XJFY6S-08	99.34	1.24	93,14
A. ferrianus MG	98.72	0	98,72

Completeness: A genome should be described as complete or finished only if it is assembled into a single contiguous sequence with no ambiguities or gaps after careful checking for errors. Contamination: Potential contamination with other species.

Table S3: List of selected genes coding sequences involved in metabolic pathways. Genes are classified by general metabolic pathways, and in case of do not have a recognized gene name, a COG or Interpro ID annotation is used

Pathway	Gene	Description
**Sulfur metabolism**		
Heterodisulfide reductase complex cluster	*hdrB*	heterodisulfide reductase subunit B, homolog
	rhd	rhodanese-like domain protein
	tusA	conserved hypothetical protein
	dsrE	conserved hypothetical protein
	hdrC	iron-sulfur cluster-binding protein
	hdrA	pyridine nucleotide-disulfide oxidoreductase
	orf2	conserved hypothetical protein
	hdrC	iron-sulfur cluster-binding protein
	hdrB	succinate dehydrogenase/fumarate reductase, C subunit
Sulfide-quinone reductase	*sqr*	sulfide-quinone reductase, putative
Cytochrome *bd* ubiquinol oxidase	*cydA*	cytochrome d ubiquinol oxidase, subunit I
	cydB	cytochrome d ubiquinol oxidase, subunit II
Cytochrome *bo*3 ubiquinol oxidase	*cyoD*	cytochrome o ubiquinol oxidase, subunit IV
	cyoC	cytochrome o ubiquinol oxidase, subunit III
	cyoB	cytochrome o ubiquinol oxidase, subunit I
	cyoA	cytochrome o ubiquinol oxidase, subunit II
Sulfur oxygenase reductase	*sor*	Sulfur oxygenase reductase
Sulfate adenylyltransferase	*sat*	sulfate adenylyltransferase, putative/adenylylsulfate kinase
Sulfur dioxygenase	*sdo*	Sulfur dioxygenase
	COG0607	conserved hypothetical protein

Thiosulfate-quinone oxidoreductase complex operon	COG0607-2	sulfur/pyrite/thiosulfate/sulfide-induced protein
	doxDA	Thiosulfate-quinone oxidoreductase, DoxD-like family protein
	COG0725	periplasmic solute-binding protein, putative
	IPR006311	Tat pathway signal sequence domain protein
	COG1275	C4-dicarboxylate transporter/malic acid transport protein
Tetrathionate hydrolase	*tetH*	Tetrathionate hydrolase
Sulfur assimilation cluster	*cysE-2*	serine O-acetyltransferase
	cysB	sulfur assimilation transcriptional regulator, LysR family
	cysE-1	serine O-acetyltransferase
	cysS	cysteinyl-tRNA synthetase
	cysD-1	sulfate adenylyltransferase, small subunit
	cysNC	sulfate adenylate transferase, large subunit/adenylylsulfate kinase
	cysQ	3'(2'),5'-bisphosphate nucleotidase
	cysJ	sulfite reductase (NADPH) flavoprotein, alpha component
	cysI	sulfite reductase (NADPH) hemoprotein beta-component
	cysH	adenylylsulfate reductase, thioredoxin dependent
	cysD-2	sulfate adenylyltransferase, small subunit
	cysN	sulfate adenylyltransferase, large subunit
	cobA	uroporphyrin-III C-methyltransferase
Sox system	*soxA*	Sulfur oxidation protein SoxA

	soxB	Sulfur oxidation protein SoxB
	soxZ	Sulfur oxidation protein SoxZ
	soxY	Sulfur oxidation protein SoxY
	soxX-2	Sulfur oxidation protein SoxX
	soxY-2	Sulfur oxidation protein SoxY
	soxZ-2	Sulfur oxidation protein SoxZ
	soxA-2	sulfur oxidation protein SoxA
	soxB-2	Sulfur oxidation protein SoxB
**Nitrogen metabolism**		
Ammonium uptake	*amt-2*	Ammonium transporter
	amt-1	Ammonium transporter
Regulators	*glnB-2*	Nitrogen regulatory protein P-II
	glnB-1	
	ntrB	Sensory histidine kinase/phosphatase
	ntrC	DNA-binding transcriptional regulator
Nitrogen fixation	*nifU*	Nitrogen fixation protein
	nifW	Nitrogen fixation protein
	nifV	Nitrogen fixation protein
	nifS/cysE-1	Nitrogen fixation protein
	nifQ	Nitrogen fixation protein
	nifX	Nitrogen fixation protein
	nifN	Nitrogenase iron-molybdenum cofactor biosynthesis protein
	nifE	Nitrogenase iron-molybdenum cofactor biosynthesis protein

	nifK	Nitrogen fixation protein
	nifD	Nitrogen fixation protein
	nifH	Nitrogen fixation protein
	draT	NAD(+)--dinitrogen-reductase
	draG	Post-translational modulation of nitrogenase activity
	nifA	Nif-specific transcriptional activator
	nifB	Nitrogenase cofactor biosynthesis protein
	nifZ_1	Nitrogen fixation protein
	nifZ_2	Nitrogen fixation protein
	nifT	Nitrogen fixation protein
	nifS/iscS-3	Cysteine desulfurase
	nifS/iscS-2	Cysteine desulfurase
	nifS/iscS-1	Cysteine desulfurase
Dissimilatory nitrate reduction	*nirD*	nitrite reductase [NAD(P)H] small subunit NirD
	nirE	nitrite reductase large subunit
	nirE-2	nitrite reductase large subunit
	narG	nitrate reductase subunit alpha
	narH	nitrate reductase subunit beta
	narI	respiratory nitrate reductase subunit gamma
	narJ	nitrate reductase molybdenum cofactor assembly chaperone
	narK	NarK/NasA family nitrate transporter
**Hydrogen metabolism**		
Group 1- Respiratory Hydrogenases	*hynL*	NiFe large subunit
	hynS	NiFe small subunit

	isp1	Iron sulfur cluster protein
	isp2	Iron sulfur cluster protein
Group 2 - Uptake Hydrogenase	*hupL/hybC*	NiFe large subunit
	hupS/hybA	NiFe small subunit
Group 3- Reversible Hydrogenase	*hoxF*	Ni/Fe hydrogenase subunit beta /Sulfhydrogenase subunit Beta
	hoxU	Sulfhydrogenase subunit gamma
	hoxY	Ni/Fe hydrogenase subunit delta
	hoxH	Ni/Fe hydrogenase subunit Alpha
Group 4- evolving Hydrogease	*hyfB*	hydrogenase 4 subunit B
	hyfC	hydrogenase 4 subunit C
	hyfE	hydrogenase 4 subunit E
	hyfF	hydrogenase 4 subunit F
Biosynthesis	*hypF*	[NiFe] hydrogenase maturation protein HypF
	hypC-1	hydrogenase assembly chaperone HypC
	hypD-1	hydrogenase expression/formation protein HypD
	hypE	hydrogenase expression/formation protein HypE
	hypA-1	hydrogenase nickel insertion protein HypA
	hypB-1	hydrogenase accessory protein HypB
	hynD	hydrogenase maturation protease
	hynH	hydrogenase expression protein, putative
	hypA-2	hydrogenase nickel insertion protein HypA
	hypB-2	hydrogenase accessory protein HypB
	hypC-2	hydrogenase assembly chaperone HypC
	hypD-2	hydrogenase expression/formation protein HypD

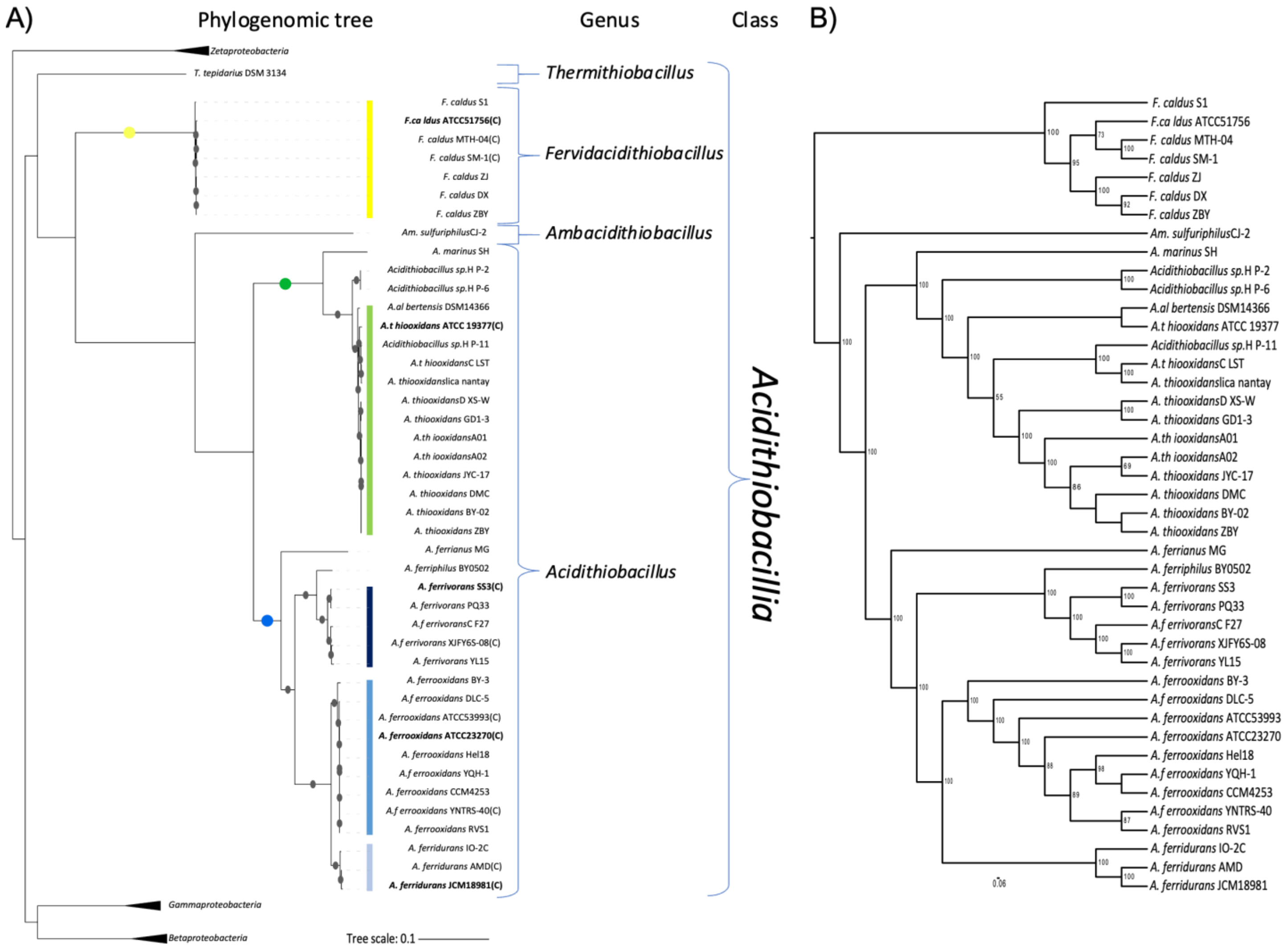

Figure S1: Phylogeny and reported phenotypes of 43 strains of *Acidithiobacillia* representatives. (A) Maximum-likelihood phylogenetic tree based on the universally shared concatenated protein sequences (core-proteins). Reference strains are shown in bold, and complete sequenced genomes "(C)". The tree highlights three major clades by colors: thermotolerant sulfur-oxidizers

(yellow), mesophilic sulfur-oxidizers (green), and mesophile/psychrotolerant iron-sulfur oxidizers (blue); (B) Dendrogram with the bootstrapping score in each intersection node, using bootstrap 1000

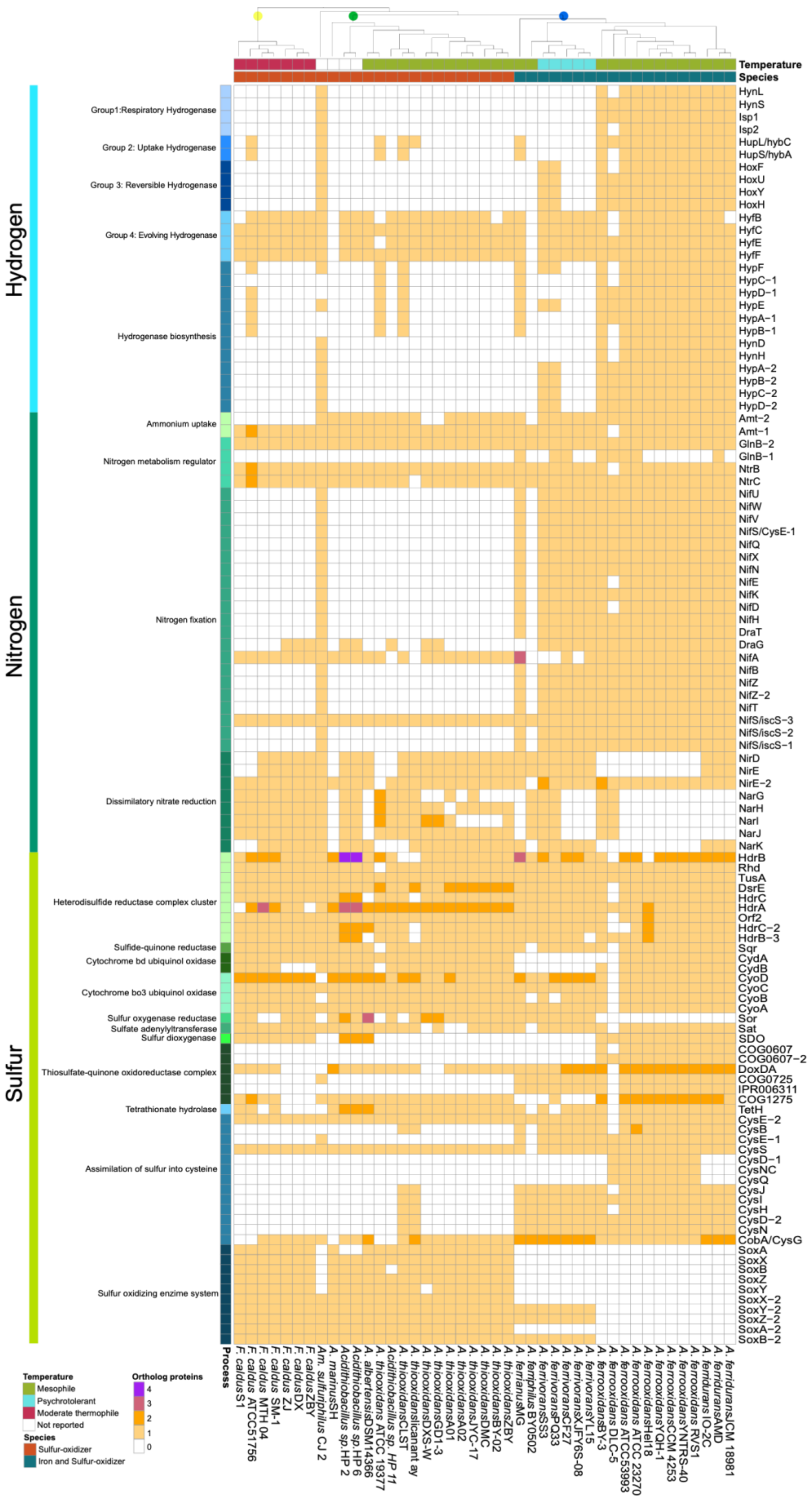

Temperature
Species
Hydrogen
Nitrogen
Sulfur
Group1:Respiratory Hydrogenase
Group 2: Uptake Hydrogenase
Group 3: Reversible Hydrogenase
Group 4: Evolving Hydrogenase
Hydrogenase biosynthesis
Ammonium uptake
Nitrogen metabolism regulator
Nitrogen fixation
Dissimilatory nitrate reduction
Heterodisulfide reductase complex cluster
Sulfide-quinone reductase
Cytochrome bd ubiquinol oxidase
Cytochrome bo3 ubiquinol oxidase
Sulfur oxygenase reductase
Sulfate adenylyltransferase
Sulfur dioxygenase
Thiosulfate-quinone oxidoreductase complex
Tetrathionate hydrolase
Assimilation of sulfur into cysteine
Sulfur oxidizing enzime system
HynL
HynS
Isp1
Isp2
HupL/hybC
HupS/hybA
HoxF
HoxU
HoxY
HoxH
HyfB
HyfC
HyfE
HyfF
HypF
HypC−1
HypD−1
HypE
HypA−1
HypB−1
HynD
HynH
HypA−2
HypB−2
HypC−2
HypD−2
Amt−2
Amt−1
GlnB−2
GlnB−1
NtrB
NtrC
NifU
NifW
NifV
NifS/CysE-1
NifQ
NifX
NifN
NifE
NifK
NifD
NifH
DraT
DraG
NifA
NifB
NifZ
NifZ−2
NifT
NifS/iscS−3
NifS/iscS−2
NifS/iscS−1
NirD
NirE
NirE−2
NarG
NarH
NarI
NarJ
NarK
HdrB
Rhd
TusA
DsrE
HdrC
HdrA
Orf2
HdrC−2
HdrB−3
Sqr
CydA
CydB
CyoD
CyoC
CyoB
CyoA
Sor
Sat
SDO
COG0607
COG0607−2
DoxDA
COG0725
IPR006311
COG1275
TetH
CysE−2
CysB
CysE−1
CysS
CysD−1
CysNC
CysQ
CysJ
CysI
CysH
CysD−2
CysN
CobA/CysG
SoxA
SoxX
SoxB
SoxZ
SoxY
SoxX−2
SoxY−2
SoxZ−2
SoxA−2
SoxB−2
Process
F. caldus S1
F. caldus ATCC51756
F. caldus MTH 04
F. caldus SM-1
F. caldus ZJ
F. caldus DX
F. caldus ZBY
Am. sulfuriphilus CJ 2
A. marinus SH
Acidithiobacillus sp. HP 2
Acidithiobacillus sp. HP 6
A. albertensis DSM14366
A. thiooxidans ATCC 19377
Acidithiobacillus sp. HP 11
A. thiooxidans CLST
A. thiooxidans Licanantay
A. thiooxidans DXS-W
A. thiooxidans GD1-3
A. thiooxidans A01
A. thiooxidans A02
A. thiooxidans JYC-17
A. thiooxidans DMC
A. thiooxidans BY-02
A. thiooxidans ZBY
A. ferrianus MG
A. ferriphilus BY0502
A. ferrivorans SS3
A. ferrivorans PQ33
A. ferrivorans CF27
A. ferrivorans XJFY6S-08
A. ferrivorans YL15
A. ferrooxidans BY-3
A. ferrooxidans DLC-5
A. ferrooxidans ATCC53993
A. ferrooxidans ATCC 23270
A. ferrooxidans Hel18
A. ferrooxidans YQH-1
A. ferrooxidans CCM 4253
A. ferrooxidans YNTRS-40
A. ferrooxidans RVS1
A. ferridurans IO-2C
A. ferridurans AMD
A. ferridurans JCM 18981
Temperature
Mesophile
Psychrotolerant
Moderate thermophile
Not reported
Species
Sulfur-oxidizer
Iron and Sulfur-oxidizer
Ortholog proteins
4
3
2
1
0

Figure S2: Phylogenetic distribution of selected proteins ortholog groups involved in assessed metabolic pathways in *Acidithiobacillia*. Heatmap revealing the presence/absence of proteins and their association with hydrogen, nitrogen, and sulfur pathways. *Acidithiobacillia* representatives are phylogenetically sorted, represented by the upside dendrogram. Based on ortholog comparison, ortholog TF counts are colored by the number (0-4) and are rearranged according to the Euclidean distance (left side dendrogram).
